# Exercise Cuts Both Ways with ROS in Remodifying Innate and Adaptive Responses: Rewiring the Redox Mechanism of the Immune System during Exercise

**DOI:** 10.3390/antiox10111846

**Published:** 2021-11-21

**Authors:** Anand Thirupathi, Yaodong Gu, Ricardo Aurino Pinho

**Affiliations:** 1 Faculty of Sports Science, Ningbo University, Ningbo 315211, China; 2 Laboratory of Exercise Biochemistry in Health, Graduate Program in Health Sciences, School of Medicine, Pontifícia Universidade Católica do Paraná, Curitiba 80215-901, Brazil; rapinho12@gmail.com

**Keywords:** physical exercise, redox balance, oxidative stress, immune system, reactive oxygen species

## Abstract

Nearly all cellular functions depend on redox reactions, including those of immune cells. However, how redox reactions are rearranged to induce an immune response to the entry of pathogens into the host is a complex process. Understanding this scenario will facilitate identification of the roles of specific types of reactive oxygen species (ROS) in the immune system. Although the detrimental effect of ROS could support the innate immune system, the adaptive immune system also requires a low level of ROS in order to stimulate various molecular functions. The requirements and functions of ROS vary in different cells, including immune cells. Thus, it is difficult to understand the specific ROS types and their targeting functions. Incomplete transfer of electrons to a specific target, along with failure of the antioxidant response, could result in oxidative-damage-related diseases, and oxidative damage is a common phenomenon in most immune disorders. Exercise is a noninvasive means of regulating ROS levels and antioxidant responses. Several studies have shown that exercise alone boosts immune functions independent of redox reactions. Here, we summarize how ROS target various signaling pathways of the immune system and its functions, along with the possible role of exercise in interfering with immune system signaling.

## 1. Introduction

Life depends on how cells effectively handle the biochemical reactions within and outside the cells (extracellular targets) [1]. This is initiated by the transfer of electrons from donor molecules—such as nicotinamide adenine dinucleotide phosphate (NAD(P)H)) and the thiol group of amino acids—to acceptor molecules, including NAD(P) and the disulfide bond of cysteine (redox) [1]. The mitochondrial electron transport chain (ETC) inescapably produces reactive oxygen species (ROS) when shuttling electrons to molecular oxygen in the interim of energy generation, thus causing increased ROS concentration and consequent damage to cellular organelles, such as DNA, proteins, and lipids [1]. In addition, other environmental oxidants—such as ozone and radiation—increase ROS generation when cells are exposed to them [1]. However, all types of cells—including myeloid and lymphoid cells—naturally produce enzymatic and non-enzymatic antioxidants that equilibrate ROS (redox homeostasis). In addition to improving the antioxidant response in immune cells, reducing equivalents in NADPH could act as cofactors in many metabolic reactions, rewiring the metabolism of immune cells [2,3]. To evade pathogens, myeloid cells—including activated neutrophils and macrophages—require these reducing equivalents as substrates for NADPH oxidase (NOX) to induce respiratory burst. A study has found that genetic alterations in these NADPH oxidase subunits can result in severe chronic granulomatous diseases (CGDs). Patients with CGDs are highly susceptible to infection [4], suggesting that changes in the redox reactions could compromise immune function.

In recent years, studies have shed light on exercise-regulated signaling pathways and the possible role of ROS in immune cells [5,6,7]. Although both the innate and adaptive immune systems are inextricably associated, exercise could be an additional factor in activating both systems via the regulation of redox-sensitive molecules by maintaining ROS within the physiological concentration. In addition, external stress caused by exercise induces mechanical stress, including shear stress; this promotes the release of stress hormones such as catecholamines and cortisol, resulting in the production of various cytokines—such as interleukin 6 (IL-6) and IL-8—and increasing neutrophil mobilization in the innate immune system, all of which cause fluctuations in ROS levels, promoting either immune function or immune suppression [8,9]. Exercise-induced IL-6 could be innately linked to acquired immune responses by promoting specific differentiation of CD4^+^ T cells [10], whereas IL-6 is involved in rebalancing energy substrate metabolism in order to improve exercise performance [11]. In addition to helping neutrophil mobilization via exercise, IL-6 activates and reorganizes various NADPH oxidase complexes to transfer electrons from NADPH to its substrate O_2_ [12]. Furthermore, exercise can activate p47phox downstream kinases, including protein kinase C (PKC), mitogen-activated protein kinase (MAPK), and protein kinase B (PKB)—also known as AKT—to induce conformational changes in the NADPH oxidase complex to interact with p22phox. Consequently, p47phox translocates to the membrane during assembly of the active NADPH oxidase enzyme complex, along with other subunits, including p67phox and p40phox [13]. This scenario could eventually transfer NADPH-derived electrons to O_2_ to produce a respiratory burst [13]. Exercise alters enzymatic kinase activity by altering redox-sensitive proteins. Wright et al. showed that hydrogen peroxide (H_2_O_2_) inhibits all phosphatase activities for increasing phosphorylation levels [14], whereas superoxide dismutase (SOD) could be an important signal in inactivating phosphatase by converting superoxide to H_2_O_2_ [15], which eventually induces conformational changes in NADPH oxidase enzyme complexes. Nevertheless, the mismanagement of exercise protocols limits these benefits.

## 2. The Roles of Acute- and Chronic-Exercise-Induced ROS in Immune Function

Depending on ROS generation, the type, intensity, and duration of exercise can significantly extend or diminish immune function. For instance, acute-exercise-induced temporary immune suppression could be linked with the increased generation of ROS. This was confirmed by the performance of acute heavy resistance exercise (squat exercise—no more than 10 repetitions for each set until the failure of the squat (80% 1-RM), for three days), during which circulating TNF-alpha was increased immediately post-exercise and 30 min post-exercise [16]. Another study reported that acute exercise in a cycle ergometer with severe intensity for up to 60 min (85% VO_2 max_) alters TNF-alpha, IL-6, and the IL-6:IL-10 ratio [17], suggesting that aerobic and resistance training at higher intensity alters cytokine levels, and showing a clear role of cytokines in increasing ROS levels, and vice versa. However, all of these factors could be returned to normal levels during the recovery or post-exercise period within 3–24 h [16,17,18,19], indicating that acute exercise (<1.5 h)-induced immune suppression does not lead to athletes being clinically deficient; rather, it increases their chances of infection [16,18], suggesting that post-exercise-induced ROS, and subsequent immune suppression, are further intensified depending on the duration of exercise carried out at higher intensity (aerobic—55–75% VO_2max_; resistance—80% 1-RM) [18]. For example, chronic exercise for a period of 24 weeks (with the intensity changing from 10 repetitions at 15RM in the first weeks to 6 repetitions at 6RM in the last week) can influence the level of cytokines [19]. One study showed that long-term intensive training for 7 months (20–25 h of pool training and 5 h of land training) suppresses immunity in well-trained athletes by reducing NK cells [18]; although this study did not evaluate the formation of ROS during or after the exercise, chronic exercise for a period of 6–7 months could possibly nurture an ROS environment for downregulating NK cell function, thus suppressing immune function. Nevertheless, chronic repetition of exercise can reduce oxidative stress via an adaptive mechanism that further supports the immune function rather than promoting ROS-induced immune suppression.

## 3. Role of Exercise-Induced ROS in T-Cell Activation

**Figure 1:**
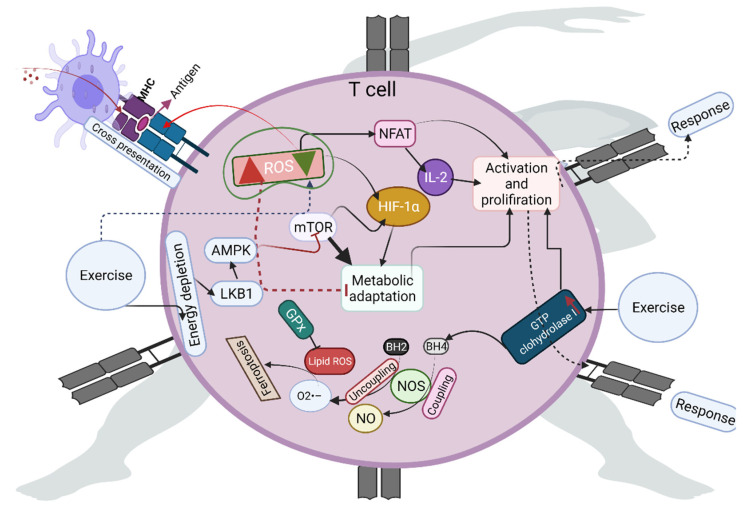
Exercise-induced reactive oxygen species (ROS) at specific concentrations promote metabolic adaptation in the T cells through the nuclear factor of activated T cells (NFAT) and mammalian target of rapamycin (mTOR), facilitating T-cell activation, proliferation, and immune response against pathogens. Exercise increases the level of GTP cyclohydrolase 1 for coupling BH4 (tetrahydrobiopterin) with nitric oxide synthase (NOS) to produce nitric oxide (NO); otherwise, it leads to ferroptosis.

The role of ROS in reshaping proteins, including enzymes and their cofactors, activates various signaling pathways in T cells [20]. This facilitates activation, differentiation, and T-cell responses. For example, ROS can act as extracellular stimulatory signals that direct the T-cell receptor (TCR) to recognize antigens on major histocompatibility complex (MHC) molecules (Figure 1). NOX- or ETC-derived ROS can act as positive feedback loops to improve TCR signaling by inactivating non-receptor tyrosine phosphatase and regulating calcium homeostasis [21,22,23]. Mitochondrial ROS facilitate the cross-presentation of antigens to trigger CD8^+^ T-cell responses. Sustained generation of low ROS levels from NOX2 can efficiently consume protons and regulate alkalinization phagosomes during cross-presentation [24]. However, higher exposure to ROS impairs the T-cell inflammatory response [2,25]. Studies have shown that optimal exercise presets cells—including immune cells—against ROS-induced damage, by activating several adaptive signaling pathways, including 5′ adenosine monophosphate-activated kinase (AMPK), nuclear factor erythroid 2-related factor 2 (Nrf-2), and nuclear factor of activated T cells (NFAT) [22,26]. In addition, these molecules and their upstream and downstream targets are connected to rewiring metabolic reactions, supporting the activation and proliferation of T cells. For example, exercise-stimulated release of Ca^2+^ from the endoplasmic reticulum can induce mitochondrial tricarboxylic acid (TCA) cycle enzymes and increase ROS to activate NFAT and subsequent IL-2 production. NFAT is a key regulator of T-cell development [27,28,29,30]. Mammalian target of rapamycin (mTOR) is implicated in T-cell homeostasis, and loss of mTOR activity impairs the development and maintenance of T cells. Exercise activates mTOR in an ROS-dependent manner by activating several molecules, including hypoxia-inducible factor (HIF-alpha) and liver kinase B1, resulting in the differentiation of naïve T cells into T helper cells [31,32]. Exercise-activated mTOR acts as a signaling node for inducing several ROS-dependent signals—such as immune receptor signaling and metabolic reprogramming—to establish an immune response. Mitochondrial biogenesis promotes CD8^+^ T-cell memory formation, metabolic fitness, and antitumor immunity, while exercise-induced ROS-dependent activation of peroxisome proliferator-activated receptor-gamma coactivator (PGC)-1alpha forces mitochondrial biogenesis [33]. AMPK-dependent ROS formation improves long-term T-cell fitness and effector/memory T-cell survival by increasing the mitochondrial membrane potential of T cells and resolving ROS-mediated toxicity [34], while energy fluctuation during exercise activates AMPK pathways. Higher levels of ROS generated during high-intensity exercise can entirely alter this scenario and stimulate immunosuppression [35].

For antioxidant functions, exercise can maintain redox homeostasis in T cells by enhancing antioxidants and the transcriptional factor Nrf-2. For example, exercise can help to replenish and maintain glutathione (GSH) biosynthesis in T cells [36,37,38]. The deficiency of antioxidants such as catalase (CAT) induces apoptosis via H_2_O_2_ in the T cells of the human immunodeficiency virus (HIV) [34]. Enhanced CAT expression in T cells reduces oxidative stress caused by activated monocytes and granulocytes in patients with chronic inflammatory conditions [39]. Furthermore, antioxidants such as glutathione peroxidase (GPx) inhibit lipid hydroperoxides in T cells; thus, they can abrogate the antigen-specific T cells by causing ferroptosis [40]. Tetrahydrobiopterin is a non-enzymatic antioxidant and a crucial cofactor for nitric oxide (NO) production. Additionally, tetrahydrobiopterin decreases superoxide production, preventing ferroptosis by phospholipid modification [41]. Exercise can increase the expression of GTP cyclohydrolase 1—the rate-limiting enzyme in the de novo biosynthesis of tetrahydrobiopterin—to decrease oxidative stress and increase the bioavailability of NO by coupling with nitric oxide synthase [42]. The deficiency of GTP cyclohydrolase 1 may decrease T-cell proliferation in autoimmunity. Resistance and aerobic exercise have been shown to influence the antioxidant pool—including SOD, CAT, and GPx—to reestablish redox homeostasis to combat ROS in HIV patients [43]. In addition, both aerobic and resistance exercise increase the number of T cells and the CD4^+^/CD8^+^ ratio, which boosts immune function in HIV [43].

## 4. Effects of Exercise-Induced Redox Homeostasis on B-Cell Activation

**Figure 2:**
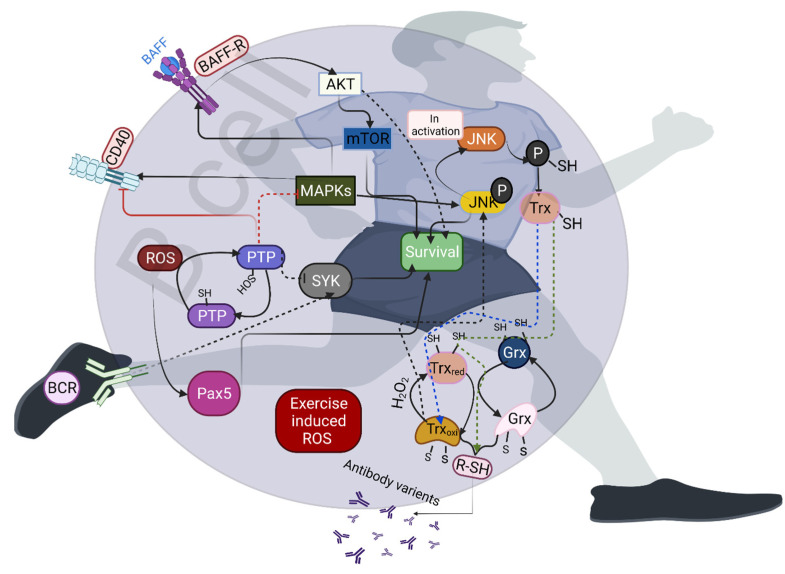
Exercise-induced ROS induce various thiol-specific targets in the B cells to produce various antibody variants. ROS-targeted protein tyrosine phosphatase (PTP) inhibits spleen tyrosine kinase (SYK) and mitogen-activated protein kinase (MAPK), while SYK and MAPK are involved in the proliferation and survival of B cells. B-cell-receptor-induced ROS activate GSK3, ERK, JNK, mTOR, and AKT for T-cell survival. Exercise-induced Trx oxidation phosphorylates JNK, leading to B-cell activation and survival.

The stimulation and proliferation of B cells rapidly induce ROS production. Thus, B cells are equipped with robust antioxidant systems; otherwise, they require enhanced antioxidant activity [1]. Sources such as NOX2 produce ROS during the early stage of B-cell activation, and mitochondrial respiration can generate ROS for later stages of B-cell activation [44,45]. However, other sources—including the oxidation of proteins and 5-lipooxygenase—also induce ROS production in B cells. Exercise influences the signaling nodes of ROS-sensitive proteins, including transcription factors, consequently influencing the early development and maturation of B cells. For example, transcription factors such as paired box (Pax5) are involved in B-cell development and maturation, and H_2_O_2_-mediated oxidation enhances the DNA-binding capacity of Pax5 [46,47]. Although no studies have reported the direct effects of exercise on Pax5, it is possible that exercise-induced H_2_O_2_ could activate Pax5 in B cells. Protein tyrosine phosphatase (PTP) is another important target of ROS, which induces reversible oxidation and further inactivation of PTPs. PTP1 negatively regulates CD40, toll-like receptor 4 (TLR4), and B-cell-activating receptor (BAFF-R) signaling in B cells. Oxidation of PTP can counteract spleen tyrosine kinase activity (Syk) to amplify B-cell receptor (BCR) signaling (Figure 2) [48]. Moreover, PTP1 regulates MAPK signaling. Studies have shown that exercise-activated MAPK and SIRT1 can directly repress PTP1, and exercise-induced ROS may be the major factors in activating such signaling [49,50]. Furthermore, p38 MAPK rapidly and transiently stimulates CD-40-dependent proliferation and negatively regulates BCR-dependent B-cell proliferation [51], while exercise-induced H_2_O_2_ mediates p38MAPK activation [52,53]. Exercise can regulate the activation of MK2 (MAPKAPK-2), which plays a crucial role in the proliferation and class-switch recombination of B cells by activating Beclin 1 [51,54]. Other signaling pathways—including glycogen synthase kinase-3 (GSK3), extracellular signal-targeted kinase (ERK), c-Jun N-terminal kinases (JNKs), mTOR, and AKT—require ROS for their activation by BCRs, including BCR and C-X-C chemokine receptor type 4 (CXCR-4) [55,56]. These signaling pathways are activated during exercise, and exercise-induced ROS could be a possible reason for the activation of such pathways.

Transcription factors such as BTB domain and CNC homolog 1 (Bach1) and Bach2 promote B-cell differentiation in various ways, including regulating class-switch recombination and plasma cell differentiation by altering mitochondrial ROS, while exercise regulates mitochondrial dynamics through ROS [57,58]. Studies have shown that ROS are required in order to activate B cells, and that ROS inhibition fails to activate and differentiate B cells [59]. Exercise-altered enzyme–substrate complexes can significantly influence the proliferation of B cells in an ROS-dependent manner. For example, thioredoxin (TRX1) can rewire metabolic reprogramming and B-cell proliferation in the bone marrow, and the absence of TRX1 can significantly induce B-cell death in tumor cells [1]. Therefore, targeting of unlocking or activating the TRX1 system can increase B-cell proliferation. Thioredoxin-interacting protein (TXNIP)—also known as thioredoxin-binding protein-2—acts as a physiological inhibitor of TRX, and plays a major role in the maintenance of metabolic functions in B cells by suppressing glucose uptake. A recent study has shown that acute exercise downregulates TXNIP in the skeletal muscle through activation of AMPK [60]. Another study showed that TXNIP promotes oxidative stress by inhibiting TRX activity [61]. In addition, exercise influences the thiol/disulfide redox state and plasma cysteine concentration [62]. This scenario generates several structural variants of antibodies with trisulfide or thioether linkages [63]. Superoxide production from NOX influences cell cycle entry, and ROS contribute to promoting BCR-dependent proliferation by negatively regulating the signaling pathways. Furthermore, NOX alters MHC class II antigen presentation by B cells [64]. Studies have shown that exercise induces NOX activity for ROS production [65,66], and that the number of B cells—including memory and naïve cells—increases after exercise [67,68].

## 5. Redox Homeostasis and Exercise in Macrophages

**Figure 3:**
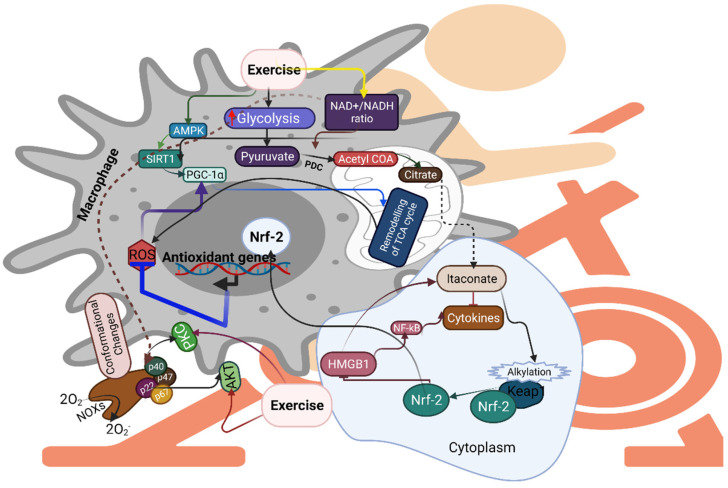
Exercise-induced NAD+/NADH ratio activates AMPK, SIRT1, and PGC-alpha in an ROS-dependent manner, and remodels TCA products such as itaconate, which controls cytokine expression and induces the alkylation of Kelch-like ECH-associated protein 1 (Keap1), facilitating the translocation of Nrf-2 into the nucleus. Exercise induces high-mobility group box 1 to increase itaconate metabolism and activate nuclear factor kappa B (NF-κB) to increase cytokine expression. Exercise activates downstream targets such as PKC and AKT to induce NOX conformational changes for superoxide production in the activated macrophages.

Exercise activates various proinflammatory responses in macrophages, in an ROS-dependent manner. For example, exercise-induced hypoxia activates several redox-sensitive proteins, including HIF-alpha, which shifts metabolic adaptation and its activation in macrophages by inducing nuclear factor kappa-light-chain-enhancer of activated B cells (NF-κB); otherwise, it impairs the metabolic shift and failure of phagocytosis [69,70,71]. Exercise causes an increase in IL-10, which has strong anti-inflammatory activity and can suppress the generation of ROS; this can further attenuate the effects of proinflammatory cytokines [72]. Exercise decreases IL-6 and F4/80 to activate SIRT-AMPK-PGC-1 alpha by suppressing NF-κB [29], suggesting the role of exercise in regulating proinflammatory response and metabolic reactions in macrophages (Figure 3). In addition, exercise-induced ROS-dependent pathways such as PGC-1alpha are linked with increased mitochondrial biogenesis, antioxidant expression, and inhibition of inflammatory pathways in macrophages [73,74,75]. Llimona et al. showed that PGC-1alpha expression is crucial for phagocytic stimulus in acute pancreatitis [76].

Studies have shown that bactericidal activity is increased in a β2-adrenergic-receptor-dependent manner in macrophages [77,78]. Exercise reduces β2-adrenergic receptors in macrophages by influencing NF-κB activation, along with β-arrestin-2 and G-protein-coupled receptors in macrophages [78,79]. In addition to mitochondrial ETC and NADPH oxidase as sources of ROS, other sources—such as pyruvate dehydrogenase complex (PDC)—can influence the NAD^+^/NADH ratio during sprint exercise [80], and proinflammatory macrophages sustain the oxidation of pyruvate via PDC for the synthesis of itaconate and cytokine production [81]. A recent study has shown that exercise-induced high-mobility group box 1 (HMGB1) increases itaconate metabolism in the TCA cycle, affecting Kupffer cells in an Nrf-2-dependent manner [82]. Itaconate alters the key glycolytic enzymes and NLRP3 inflammasome, and activates Nrf-2 via the alkylation of Kelch-like ECH-associated protein 1, resulting in the inhibition of proinflammatory responses in M1 macrophages [83]. Activation of Nrf-2—either genetically, or by a chemical inducer—alters the proinflammatory response in macrophages [84]. Furthermore, pyruvate dehydrogenase kinase (PDC1) is a key enzyme that regulates glucose metabolism in macrophages. A study has shown that knockdown of PDC1 reduces M1, whereas it increases M2 activation by regulating glycolysis and glucose oxidation [85]. Prolonged physical activity induces hypomethylation of genes involved in PDC [86]. In summary, exercise can influence the ROS-sensitive transcription factors and the NAD^+^/NADH ratio, and can rearrange metabolism in an ROS-dependent manner.

## 6. Role of Exercise-Induced ROS in the Activation of Immune Receptors 

**Figure 4:**
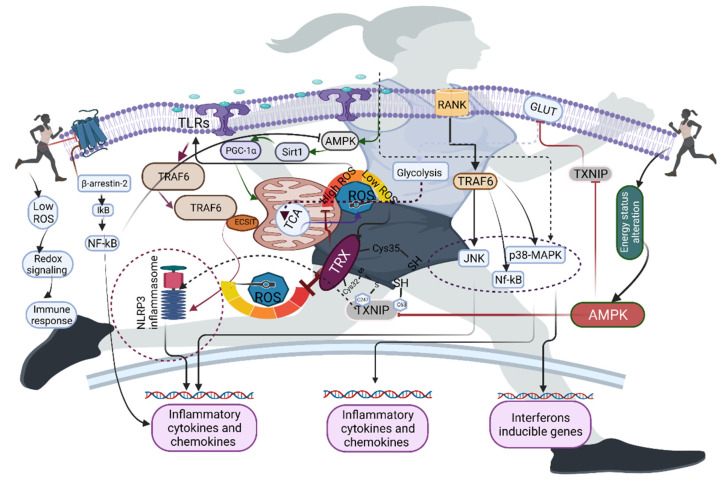
Exercise-induced ROS activates immune receptors such as toll-like receptors (TLRs), receptor activator of NF-κB (RANK), and beta-adrenergic receptors. Exercise-induced ROS at limited concentrations activate the TLRs and their target TRAF6, inducing the NLRP3 inflammasome in an ECSIT-dependent manner. Exercise-induced energy status alteration can inhibit TXNIP via AMPK, which further inhibits high ROS levels via TRX. RANK-induced TRAF6 activates inflammatory cascades via JNK, NF-κB, and p38-MAPK, whereas exercise inhibits the β-adrenergic receptor to generate a feedback loop in the immune cells in order to regulate the inflammatory cascades.

Immune receptors are sensitive to ROS, and low levels of ROS can activate the Nod-like receptor (NLR) family, TLRs, and cytosolic DNA sensors, whose function is to recognize the molecular patterns of pathogens and damaged tissues of the host in order to evade them by producing cytokines and interferons. For example, TLRs—particularly TLR4—in neutrophils require ROS for their activation [87]. TLR4 is activated for the formation and maturation of cytokines and interferons via the activation of NF-κB. Indeed, acute exercise with higher intensity or prolonged duration activates NF-κB in an ROS-dependent manner [87]. Another specialized group of receptors—NLRs—recognize microbes and other danger signals by activating and releasing cytokines and interferons [88]. TXNIP is linked with the activation of NLRP3, and TXNIP is an inhibitor of TRX, suggesting that inhibition of TRX could increase ROS generation to activate NLRP3 (Figure 4). A study has shown that the NLRP3 activator can induce the formation of ROS [89]. As mentioned above, an acute bout of exercise decreases TXNIP in an AMPK-dependent manner [60], possibly because an acute bout of exercise may facilitate NOX2-induced ROS that increase TXNIP. However, there is a lack of evidence on how and what sources of ROS could facilitate NLRP3. Although studies have shown that the NOX2 complex may be one of the reasons for this NLRP3 activation, NOX2 deficiency in mice does not affect inflammasome activation [90], suggesting that other sources of ROS may contribute to the activation of NLRP3.

In addition to sensing for activation of the receptors, ROS can also act as secondary messengers to regulate various immune functions [91,92]. For example, receptor activator of NF-κB ligand (RANKL) and its receptor RANK induce recruitment of TRAF6 to the cytoplasmic domain, activating various signaling pathways, such as MAPK, JNK, and p38MAPK. Exercise-induced ROS can act as secondary messengers for activating those signaling pathways [87,93,94]. Studies have shown that TRAF6 deficiency blocks RANKL-mediated formation of ROS, and further impairs JNK, MAPK, and ERK signaling [95]. RANK-L/osteoprotegerin (OPG-L) and RANK play crucial roles in regulating immune function; this was established with RANK-L/OPG-L-deficient mice, which have diminished thymic cellularity size [96,97]. Moreover, RANK-L/OPG-L-deficient mice have impaired maturation of CD4+ and CD8+ in the thymus [96,97], suggesting that RANK-L/OPG-L is a crucial factor for T-lymphocyte maturation in the thymus. Furthermore, ROS play a crucial role in intracellular bactericidal activity—especially mitochondria-generated ROS—as opposed to NADPH oxidase, which generates ROS for phagosomes [98,99]. TLRs—including TLR1, TLR2, and TLR4—recruit mitochondria to macrophage phagosomes and augment ROS production [98]; this is achieved via translocation of a TLR signaling adaptor and TRAF6 to the mitochondria, where they interact with evolutionarily conserved signaling intermediates in the toll pathway (ECSIT) [98]. It has been established that ECSIT and TRAF6 deficiency can decrease TLR-induced ROS, impairing the killing of intracellular bacteria [98]. A study has shown that increased expression of antioxidant enzymes such as CAT, and subsequent decrease in ROS, can impair bacterial activity [98].

## 7. Exercise in Activating Neutrophil Extracellular Traps (NETs)

The formation of neutrophil extracellular traps (NETs) is a crucial strategy for protection against infection. Exercise affects NET pathways at various levels in an ROS-dependent manner. For example, exercise-activated ERK signaling reassembles NOX, while activation of protein-arginine deiminase 4 (PAD4) is crucial for the citrullination of histones H3 and H4; this further activates neutrophils to induce the release of NETs [100]; exercise-induced ROS could be a possible reason for this phenomenon. A study showed that acute severe exercise improved the formation of NETs by increasing NOX-induced ROS and reducing mitochondrial membrane potential [101]. In addition, regular endurance exercise comprising more than 5 h/day facilitated the release of NETs, which further impacted the innate defense in an ROS-dependent manner [101], suggesting that NET formation can occur relative to the training loads in highly trained athletes [102]. In contrast, exhaustive exercise-induced lactic acid accumulation alters this scenario, independent of ROS formation, and inhibits the formation of NETs; this may be due to metabolic flexibility caused by exercise, and understanding metabolic checkpoints with different exercise modes will offer new perspectives for developing and targeting unregulated NET-mediated diseases [103,104].

## 8. Therapeutic Opportunities and Future Directions for Exercise-Induced ROS

Exercise-induced ROS can provide various therapeutic targets that shield against immune disorders. In particular, ROS-sensitive signaling and its downstream targets are obvious earmarks of exercise-induced ROS [99]. Changes in ROS levels increase T-cell activity under autoimmune conditions. Exercise-induced metabolic shift favors a reductive environment in immune cells, which results in the maintenance of low levels of ROS; this may increase T-cell activity robustly under autoimmune conditions [99]. Therefore, exercising to increase ROS signaling or presetting adaptive responses can alleviate autoimmune conditions. Restoring ROS signaling reduces inflammation in rheumatoid arthritis, in contrast to the conventional view of ROS increasing tissue inflammation in rheumatoid arthritis, which suggests that exercise can retune the redox system and influence the functional capacity of immune cells [105]. Deficiency in Nrf-2 activation is linked to several autoimmune conditions. A recent study has shown that Nrf-2 deficiency promotes lupus nephritis with altered Th17 activation [105]. Activation of Nrf-2 reduces autoimmune inflammation independently of regulatory T-cell dysfunction in Scurfy mice [106]. Depending on type, intensity, and duration, exercise maintains ROS at a physiological concentration to activate Nrf-2 signaling [107,108]. Another possible therapeutic target is AMPK, which is linked with exacerbation of autoimmune diseases. Evidence has shown that loss of AMPK activity aggravates autoimmune encephalomyelitis [109], and exercise is a well-known activator of AMPK. However, achieving a successful therapeutic target in autoimmune conditions requires solid exercise protocols, which may establish optimal ROS environments in each stage of immune cells.

## 9. ROS-Mediated Clinical Evidence of Immunity Changes in Athletes

Exercise mode influences transient immune suppression, and ROS can be major mediators of this event. Studies have shown that acute exercise increases upper respiratory tract infections (URTIs) [110,111], but these symptoms were decreased in well-trained athletes during marathon or ultramarathon events, or even heavy training [111]. However, the specific rationale is not well established, and a possible reason for this is redox homeostasis perturbation and further increase in inflammatory cytokine levels [112,113]. This is evidenced by the increase in anti-inflammatory cytokines, NK cells, and neutrophils in the circulation during moderate-to-vigorous exercise (<60 min), and these factors play a crucial role in the fluctuation of ROS levels, and have important clinical value in normal and diseased individuals [112,114,115,116]. For example, moderate exercise decreases the incidence of infection when compared to either higher intensity with marked load training or physical inactivity [117]; this may be due to transient immune competence, which takes place in athletes for several hours (3 and 72 h), facilitating a so-called “open window“, which implies that diminished immune function increases the risk of clinical infection after exercise [117]. In this scenario, higher levels of inflammatory reactions and further parallel activation of neutrophils and macrophages can increase ROS-induced immunosuppression [118]. In addition, understanding of immune response in males and females during exercise could promote the optimization of athletic performance and improve health [118]. Studies have shown that proinflammatory cytokines are significantly increased after exercise in the luteal phase of females, compared to males [119]. Furthermore, a study has shown that 90 min of cycling at 65% maximum aerobic power increases the levels of neutrophils, monocytes, and lymphocytes during the luteal phase of the menstrual cycle compared to the follicular phase [120], suggesting that the immune system can undergo several changes determined by sex-specific responses to exercise.

## 10. Conclusions

This review summarizes the current knowledge of exercise-induced ROS in regulating functional changes in immune cells. Although exercise orchestrates redox homeostasis for rewiring metabolic responses in immune cells—including T cells, B cells, and macrophages—an acute bout of exercise with increased intensity alters this scenario, and facilitates an open window for further infection. In contrast, prolonged exercise maintains immune competence for several hours, during which a possible ROS environment could provide pre-adaptive settings against oxidative stress and activate various molecular signaling pathways to promote immune function after exercise. However, this should be established based on specific ROS formation according to the duration of the specific exercise, which would help to identify the exact duration induced by ROS to promote immune functions. In addition, the impact of exercise on stimulating innate immune function by activating NOX-induced ROS is obvious. However, investigating how this is effectively transferred to adaptive immunity, and identifying the specific molecular targets that are more closely involved in carrying out this function in an ROS-dependent manner, will further the use of exercise as a novel therapeutic strategy to increase both innate and adaptive immune responses against various infections.
